# Heavy chain dimers stabilized by disulfide bonds are required to promote in vitro assembly of trastuzumab

**DOI:** 10.1186/s12860-019-0244-x

**Published:** 2020-01-21

**Authors:** Mercè Farràs, Ramón Román, Marc Camps, Joan Miret, Óscar Martínez, Xavier Pujol, Antoni Casablancas, Jordi Joan Cairó

**Affiliations:** 1 Department of Biotechnology, Farmhispania SA, Montmeló, Spain; 2 Department of Chemical, Biological and Environmental Engineering, Autonomous University of Barcelona, Cerdanyola del Vallès, Spain

**Keywords:** mAb, Protein structure, Folding, Disulfide bonds, Renaturalization, LC, HC, mAb assembly, Urea, Anti-HER2, Non-covalent, Slow dialysis, Glutathione, 2-mercaptoethanol, Affinity chromatography, Trastuzumab, Immunoglobulin

## Abstract

**Background:**

Monoclonal antibodies (mAbs) and their derivatives have become one of the most important classes of therapeutic drugs. Their multiple applications increased the interest for understanding their complex structure. In vivo, animal cells are able to fold mAbs correctly (Song et al, J Biosci Bioeng 110:135-40, 2010), whereas previous in vitro approaches were scarce and mostly unsuccessful.

**Results:**

In this work, we compared in vitro assembly characteristics of trastuzumab, produced either by A) physical separation and refolding of its sub-units or B) direct joining of individually produced heavy and light chains. Native and denatured structures of trastuzumab were determined by SEC-HPLC, HIC-HPLC and SDS-PAGE.

**Conclusions:**

Our results demonstrate the requirement of correctly folded HC, forming disulfide-bonded dimers, in order to form a fully functional mAb. Otherwise, the unfolded HC tend to precipitate. We were able to assemble trastuzumab in this fashion by only mixing them to LC in pH-buffered conditions, while monomeric HC structure was too unstable to render a functional mAb. This approach has been used in the generation of homogeneous ADC, with results pending to be published.

## Background

Therapeutic antibodies have risen to prominence over the past three decades and are the fastest growing drug class, with currently more than 70 monoclonal antibodies (mAbs) approved by FDA and EMA [1–3]. In view of the side effects and limitations of mAbs [4] several improvements and modifications such as conjugated mAbs fragments or bispecific antibodies [5, 6] have been developed [7]. Some of its applications include the treatment of infectious and non-infectious diseases such as cancer, immune diseases, arthritis and other disorders resulting from organ transplantation [8].

### Mabs in vivo structure

The complex structure of mAbs lead to further efforts in order to understand their folding, denaturation and refolding [9, 10]. In vivo, heavy chains (HCs) and light chains (LCs) are co-translationally translocated into the endoplasmic reticulum (ER) and folding begins even before the polypeptide chains are completely translated. Most immunoglobulins G (IgGs) assemble first as HC dimers to which LCs are added covalently via a disulfide bond between the CL and CH1 domains [11]. The Ig fold is characterized by a greek-key β-barrel topology in which the barrel is not continuously hydrogen bonded, but instead composed of two sheets, forming a sandwich-like structure [11].

### mAbs denaturation and refolding

Pioneering studies on antibody folding were executed on denatured LCs, allowing in vitro refolding [12]. Cold denaturation studies using guanidine hydrochloride were performed and revealed that mAbs have a potential to undergo cold denaturation at storage temperatures near − 20 °C (pH 6.3), and this potential needs to be evaluated independently for individual mAbs [13]. This previous data shows that it is possible to refold a reversible denatured mAb without breaking disulfide bonds.

The structure of antibodies and many antibody fragments is composed of multiple domains, often connected by inter-domain disulfide bonds, which further complicates the already difficult refolding processes.

Refolding of the denatured and reduced immunoglobulins is scarcely successful *in vitro* [14]. Fab fragments consisting of two subunits have been vigorously employed as model systems for studying the mechanisms of protein folding [15, 16], and many refolding technologies have been used for these fragments, comprising dilution, dialysis, solid phase solvent exchange and size exclusion chromatography [16], but several complications are observed during refolding, which have been overcome. The folding yield of the denatured and reduced Fab fragment was low by spontaneous renaturation, but in the presence of a GroE system (GroEL, GroES and ATP) or protein disulphide isomerase (PDI), the folding yield of the denatured and reduced Fab fragment was higher than that of spontaneous renaturation [17]. A similar approach using immunoglobulin heavy chain binding protein (BiP) and PDI was successful in a complete mAb [18].

### In vivo and in vitro LC and HC (re)folding

Studies regarding slow dialysis without the assistance of chaperone were performed to renature a denatured and reduced IgG at a concentration of 1 mg/ml [19] with a 70% of folding yield.

In this work, we based our In vitro refolding strategy in this slow dialysis method but adding a physical separation step by size exclusion chromatography under denaturing conditions. The main challenge was the physical chains separation and their reversible refolding due to the mAb complex structure, formed by covalent (disulfide) and non-covalent (ionic, hydrogen bonds, Van der Waals, hydrophobic) interactions to maintain the correct conformation, which is essential to revert the original mAb structure and functionality.

We studied the feasibility to unfold, physically separate mAb chains, in vitro correctly refold them and then reassemble the original anti-HER2. This assembly approach is compared with the direct reassembly of the mAb using in vivo folded chains (independently produced in HEK293 cultures). The differences between in vivo and in vitro folded chains are analyzed, as well as the impact on mAb assembly efficiency.

## Results

### Unfolding and folding a mAb without physical separation

Firstly, we adapted the Maeda et al. method [20] based on slow dialysis and appropriate redox buffer in order to test the ability of the method to refold and reoxidize denatured and reduced trastuzumab, without physical chain separation.

**Fig. 1:**
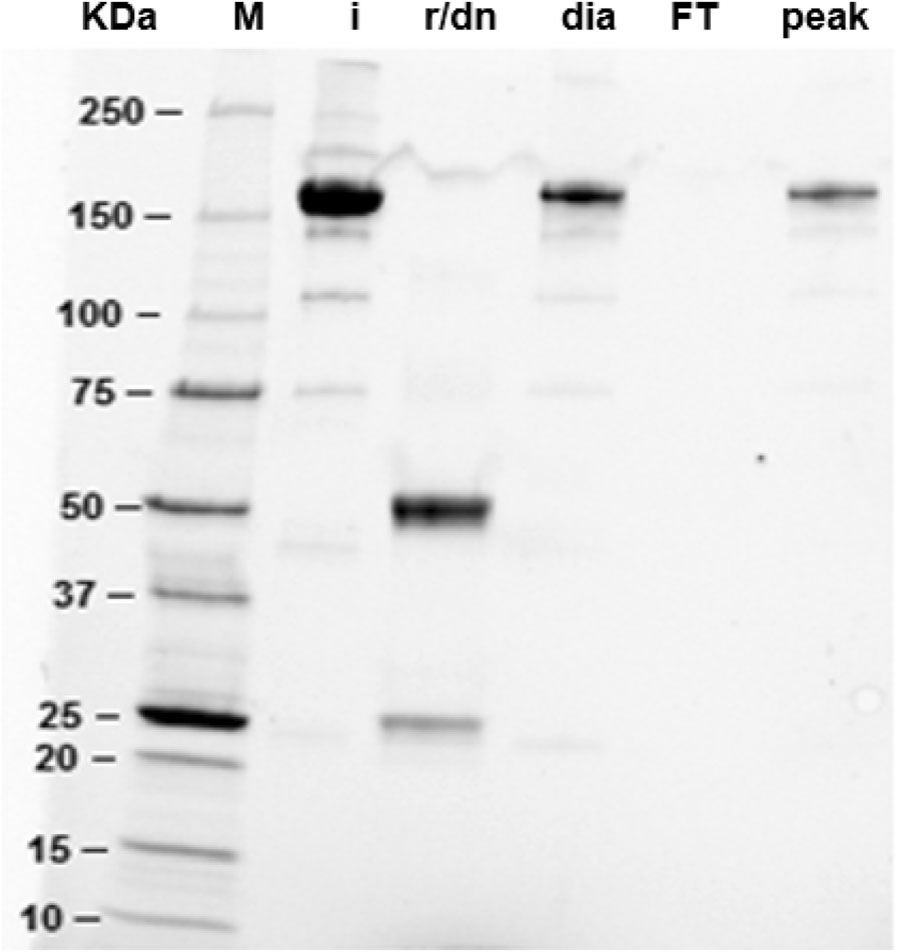
SDS-PAGE of reduced and refolded anti-HER2. M: molecular weight marker; i: intact mAb; r/dn: reduced and denatured mAb; dia: mAb dialyzed by slow dialysis; FT: *flow-though* of the affinity chromatography MAb Select SURE; peak: elution peak of the affinity chromatography

Results obtained are shown in Fig. 1, where complete reduction and denaturation of anti-HER2 is achieved in the conditions discussed (and checked by SDS-PAGE and SEC-HPLC). After slow dialysis, the antibody is able to recover its disulfide bonds, showing the same profile as the initial mAb in SEC-HPLC (results not shown). Refolded trastuzumab effectively recognizes isolated HER2 antigen in an ELISA test in the same levels as untreated control (Table 1) and binds to protein A affinity column (Fig. 1), proving that the fragment crystallizable region (Fc) is also correctly folded. Denatured and reduced mAb showed no antigen recognition in the ELISA test (Table 1).

**Fig. 2:**
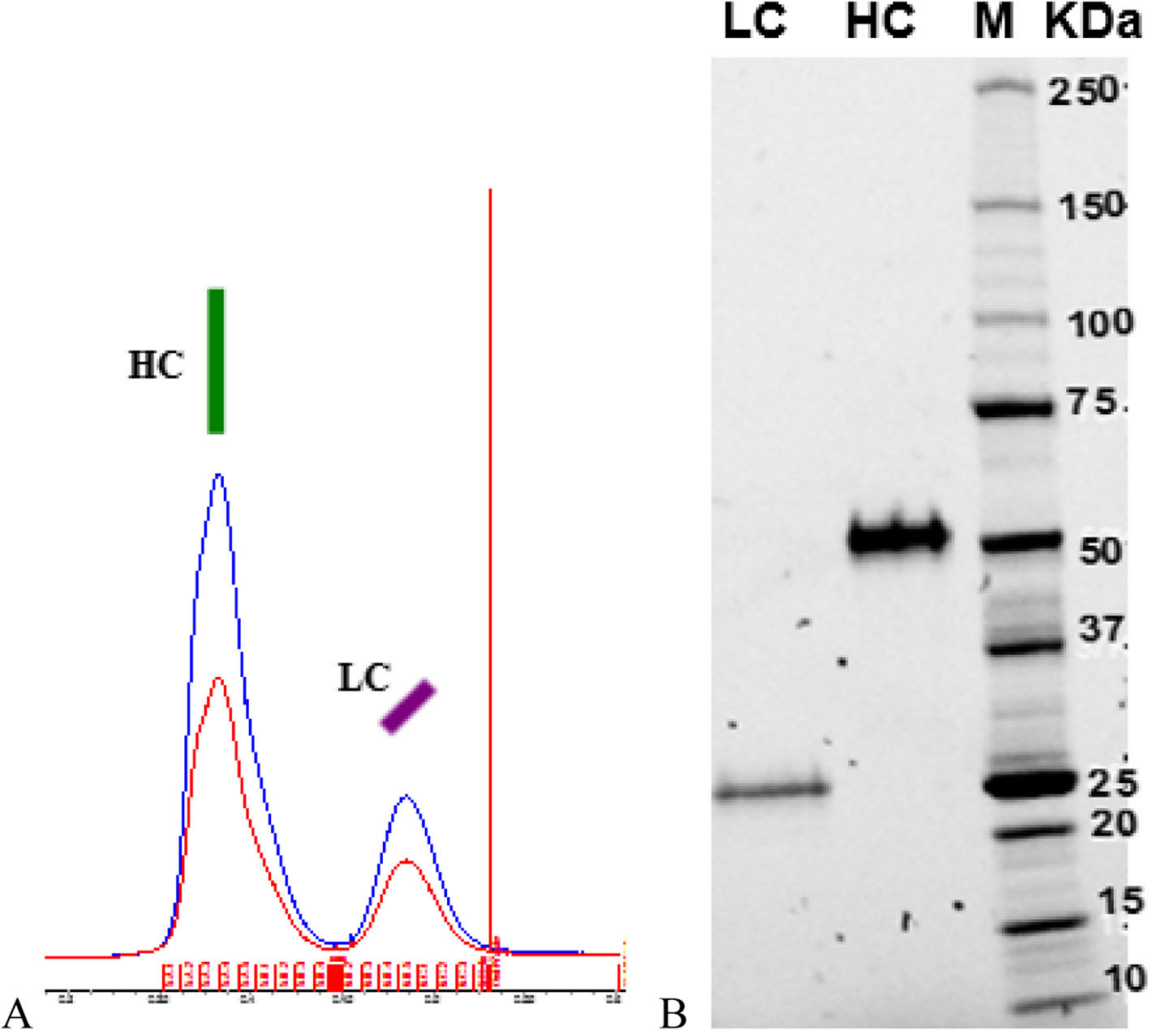
mAb chains separation with exclusion size chromatography in ÄKTA Avant system under reducing and denaturing conditions. **a**. UNICORN Software chromatogram, where absorbance at 280 nm is shaded in blue and absorbance at 260 nm is shaded in red. **b**. SDS-PAGE gel corresponding with the isolated peaks collected in the chromatography

**Table 1:** Isolated antigen HER2 recognition in the ELISA test to assess the mAb folding without chains physical separation

Molecule	Trastuzumab	Denatured trastuzumab	Renatured trastuzumab
Isolated antigen HER2 recognition	51 ± 5 AU/μg	ND	22 ± 1 AU/μg

### Unfolding and folding anti-HER with chains physical separation

Anti-HER2 interchain disulfide bonds were totally reduced and mAbs chains were physically separated and purified by denaturing SEC (Fig. 2a) as described in methods section (Fig. 8). Protein identity of each peak was validated through an SDS-PAGE gel (Fig. 2b) and confirmed the selective chains separation, with little or none cross-contamination.

After a 5 times volume reduction with a 3KDa Amicon device, slow dialysis was performed as described before for HC and LC.

**Fig. 3:**
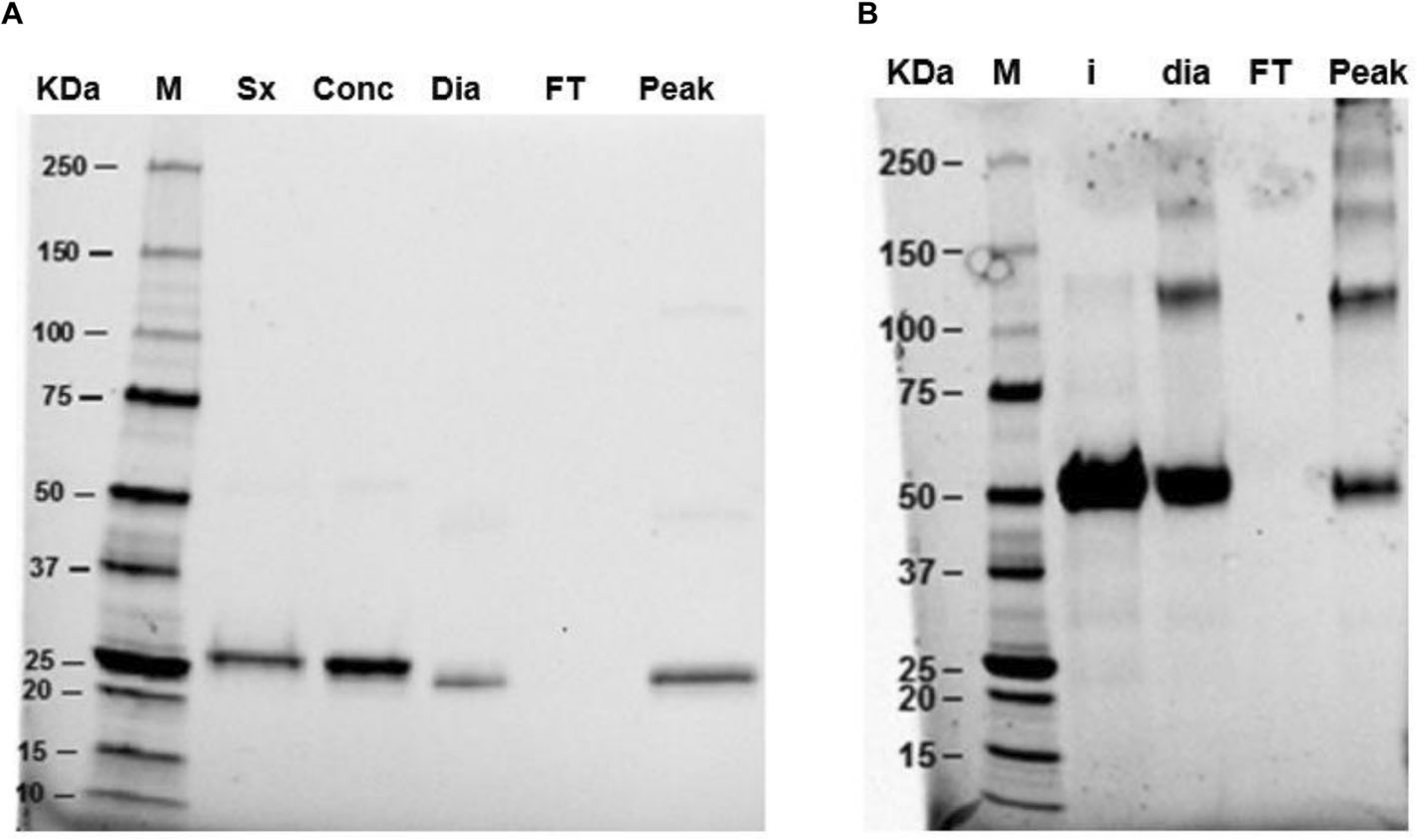
Gel SDS-PAGE of refolding sequence of LC and HC. **a**. LC refolding process. M: molecular marker; Sx: Superdex peak of denatured and reduced LC; Conc: concentrated; Dia: LC diafiltered by slow dialysis; FT: *flow-through* of Capto L; Peak: Capto L elution peak. **b**. HC refolding process. M: molecular marker; i: Superdex peak of denatured and reduced HC; dia: HC diafiltered by slow dialysis; FT: *flow-through* of MAb Select SURE; peak: MAb Select SURE elution peak

LC structural integrity was first checked by Capto L affinity chromatography and no protein could be detected in the fraction corresponding to *flow-through*, showing that reassembled LC could interact with protein L. The yield from denatured and reduced LC to the folded LC was 26% (Fig. 3a). The same approach was followed with HC by using protein A affinity chromatography to check proper binding. In this case, the fraction corresponding to the elution peak showed major aggregation in the non-reduced SDS-PAGE gel (Fig. 3b).

LC and HC were independently buffer-exchanged using PD Desalting G-25 column to exchange the elution buffer for 50 mM citrate pH 6, in order to recover the original mAb structure. Under these pH conditions, HC precipitated almost completely and the antigen recognition of the renatured trastuzumab is reduced to the half compared to the reference mAb (Table 1), indicating that the mAb structure was not recovered completely.

### In vitro and in vivo LC folding structure comparison

**Fig. 4:**
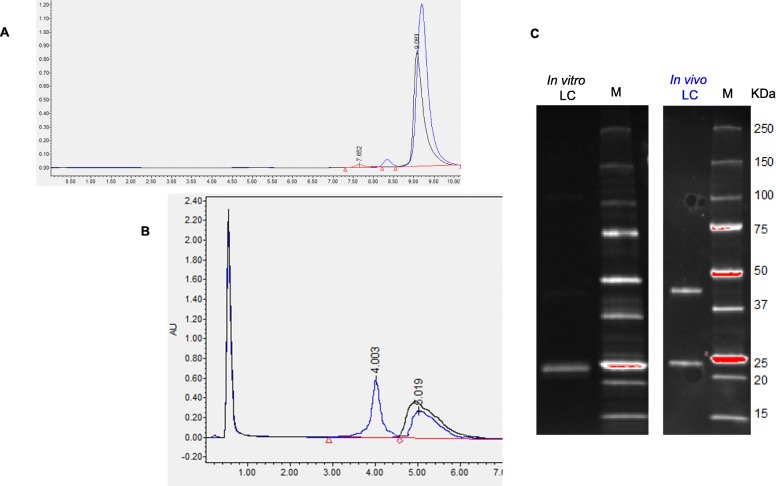
LC in vitro and in vivo structure comparison. **a**. SEC-HPLC of in vitro refolded LC (black) and in vivo folded LC (blue). **b**. HIC-HPLC of in vitro refolded LC (black) and in vivo folded LC (blue). **c**. Gel SDS-PAGE of in vitro refolded LC (black) and in vivo folded LC (blue)

LC refolded by slow dialysis (in vitro refolding) under denaturing, non-reducing conditions (Fig. 4c LC A) shows a single 21 KDa band, corresponding to a monomer structure. However, under native conditions (Fig. 4a), a molecule of 42 KDa is detected, indicating that LC is folded forming dimers without covalent interactions between them through disulfide bonds.

**Fig. 5:**
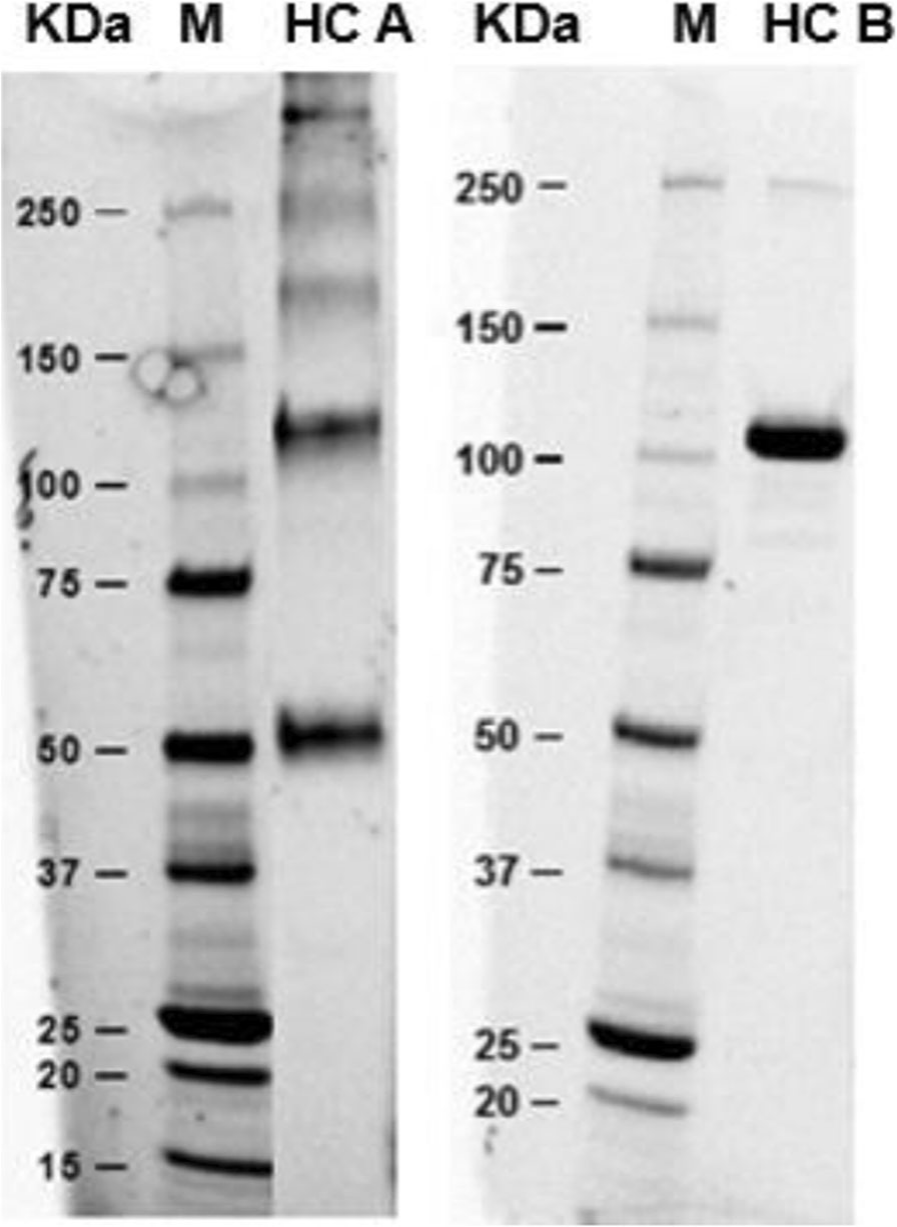
HC in vitro and in vivo structure comparison. Gel SDS-PAGE of in vitro refolded HC (HC A) and in vivo folded HC (HC B)

LC produced by HEK293 (in vivo folding), however, appears in two forms under denaturing conditions: 55% of 42 KDa band and 45% of 21 KDa band (Fig. 5c LC B). Under native conditions, 100% of LC is detected as dimers (Fig. 4a).

Hydrophobic differences were also detected: meanwhile in vitro refolded LC shows a single peak in HIC-HPLC, in vivo folded LC appears as 2 peaks (Fig. 4b). This result could be related to hydrophobicity differences between covalently and non-covalently bonded dimers, due to the structural differences among them.

LC is not able to recognize HER2 antigen alone (Table 2).

**Table 2:** Isolated antigen HER2 recognition in the ELISA test to assess the mAb folding with chains physical separation

Molecule	In vivo folded LC	In vivo folded HC	Refolded trastuzumab
Isolated antigen HER2 recognition	ND	ND	54 ± 6 AU/μg

### In vitro and in vivo HC folding structure comparison

In vitro refolded HC is mostly appearing as a monomer, with little dimers forming disulfide bonds, but covalent aggregation is also observed in SDS-PAGE gel (Fig. 5 HC A).

However, in vivo folded HC is produced forming dimers covalently linked by disulfide bonds (Fig. 5 HC B).

When in vitro refolded HC was dialyzed to pH 6, severe precipitation is observed. In vivo folded HC, under the same conditions, remained soluble.

### mAb refolding of independently produced LC and HC

In vivo folded LC and HC were dialyzed with 50 mM citrate pH 6 and mixed with a proportion of 5.54 g dcHC / g LC. Under these conditions, it is possible to recover a fully functional mAb structure without the need of slow dialysis or additional chaperones.

**Fig. 6:**
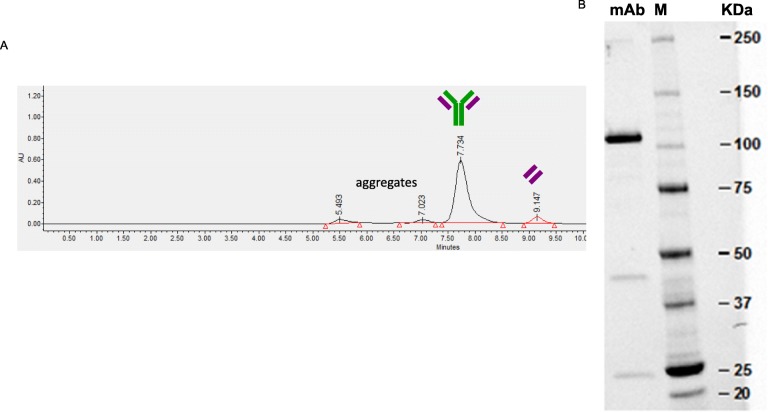
mAb refolded from independently produced LC and HC. **a** SEC-HPLC (native structure). The identity of the peaks was checked by LC-MS (data not shown). **b** Gel SDS-PAGE (denatured structure)

The final mAb effectively recognizes isolated HER2 antigen in an ELISA test in the same levels as original anti-HER2, while in vivo folded LC or HC alone do not. The assembled mAb forms a monomer through non-covalent interactions, but it was not stabilized through covalent disulfide bonds because under denaturing conditions only HC dimers (100 KDa), LC dimers (42 KDa) and LC monomers (21 KDa) are detected (Fig. 6b), probably because interchain thiols are capped with cellular glutathione and/or cysteine originated during the cell culture [21]. However, mAb activity has completely been recovered, as analyzed in the ELISA test (Table 2). The HC dimers, LC and refolded mAb identity was confirmed by LC-MS (data not shown).

## Discussion

In this work, we have observed that a denatured and reduced mAb analysed by SDS-PAGE and HPLC could be satisfactory refolded in vitro if HC and LC were not physically separated. However, if the same protocol was applied with physical separation of HC and LC followed by the refolding of each chain independently, HC showed precipitation and, therefore, the original mAb structure could not be recovered. In vivo, HC is stabilized through disulfide bonds, to which LC are added [7, 11]. Here we hypothesize, that in vitro HC needs LC interaction to generate dimers and if it appears as a monomer form, HC is so unstable that smooth pH changes promote its precipitation. It is, therefore, a requisite to have a stable HC dimer, even if it is only stabilized by non-covalent interactions [22] as in the case of DAR 7–8 Antibody Drug Conjugates, to promote mAb assembly.

These observations are supported by results obtained when HC and LC were independently produced in HEK293 cultures (in vivo*).* HC was produced as a covalently bonded dimer and their mixture to dimer LC allowed obtaining a functional mAb conformation. In this approach, it has been proved that no HC or LC reduction is required to remove glutathione from the capped thiol groups because interactions between LC and HC are non-covalent. We hypothesize that the antibody monomer form is biologically more favorable than LC dimers, which are so unstable that the reaction is displaced to bond LC to HC instead of maintaining LC-LC (even if they are covalently linked). The reduction and reoxidation of mAbs disulfide bonds have been studied in detail for manufacturing processes [23, 24] and it has been demonstrated that after partially reducing the mAb, the original disulfide bonds were reformed because the re-oxidized state is favored [25]. However, the current work indicates that the antibody form is more stable than LC dimers even if the mAb structure is not stabilized by covalent interactions.

## Conclusions

**Fig. 7:**
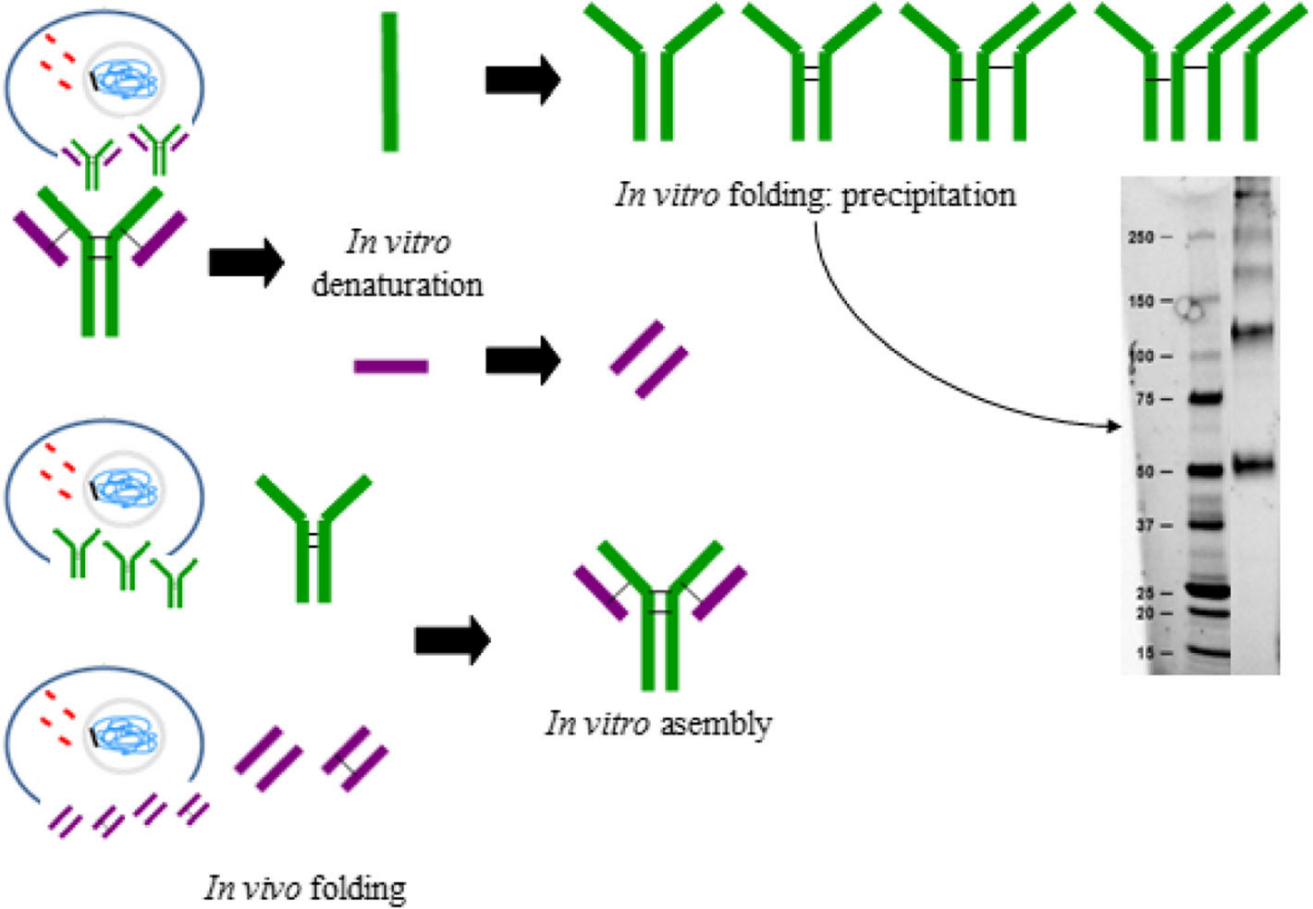
Graphical conclusions. Antibody folding is a complex process which occurs in vivo and where light chains are coupled to previously assembled heavy chain dimers. In this work, denaturation and chains separation of the Trastuzumab antibody followed by their in vitro refolding through slow dialysis method has shown that heavy chain dimers stabilized by disulphide bridges are necessary in order to reassemble the whole antibody. Successful in vitro assembly of heavy and light chains has been achieved when the chains have been independently produced

In this work we revealed that HC dimers are imperative for LC assembly in order to obtain a functional mAb structure in vitro. If the individual subunits are not correctly or partially folded before mAb reassembly, the functional mAb cannot be obtained (Fig. 7). Also, the proportion of LC and HC only affects the yield of the refolding process if a subunit is added in excess while the protein concentration could affect the refolding kinetics. Future work will study the optimization of the mAb refolding process.

## Methods

### Cell lines and maintenance

Anti-HER2 antibody sequence was obtained by synthetic nucleotide synthesis (GenScript) and cloned in a tricistronic expression vector derived from the commercial pIRESpuro3 vector (Clontech) between NheI-AgeI (LC) BamHI-EcoRI (HC).

HEK293 cells were kindly provided by Dr. A. Kamen (National Research Council of Canada). HEK293SF-F6 cells were then transfected with the construct and selected as described previously [26]. The new anti-HER2 antibody producer cell lines were referred to as HEK293_T10.

HEK293SF-3F6 producing anti-HER2 HC or anti-HER2 LC were obtained by cloning the synthetic sequences in the expression vector pIRESpuro3 (Clontech) between BamHI and NotI restriction sites for HC and in the vector pIRESneo3 (Clontech) between NheI and AgeI sites for LC. HEK293 cells were then transfected with the different constructs and selected as described previously [27]. The new producer cell lines were referred to as HEK293_THC and HEK293_TLC.

Cells were subcultured at 0.3 × 10^6^ cell/mL three times per week to keep them in exponential growing phase. Cell maintenance was performed in 125 mL polycarbonate shake flasks (Corning), with a working volume of 12 mL, and maintained at 37 °C in an incubator with a 5% CO_2_ humidity saturated atmosphere (Steri-cult 2000 Incubator, Forma Scientific). Flasks were continuously agitated at 110 rpm on an orbital shaking platform (Stuart SSL110).

### Anti-HER2, HC and LC production

Culture media used for HEK293_T10 cell line was SFM4Transfx-293 (HyClone, SH30382.00) supplemented with 4 mM GlutaMAX (Gibco, 35,050,061), 10% (v/v) Cell Boost 5 (60 g/L solution) (HyClone, SH30865.01), 2% (v/v) Kolliphor P188 (100 g/L solution) (Sigma, 15,759) and 0.5% (v/v) Antifoam C (10 g/L solution) (Sigma, A6832). Selection pressure was maintained during the production step by adding 0.2% (v/v) puromycin (1 mg/mL) (Merck, 58,582).

Bioreactor cell cultures were performed using Flexsafe RM 10 L bags (Sartorius, DFB010L) in a WAVE 20/50 EHT (GE) with a working volume of 5 L. Temperature was set at 37 °C. Agitation and angle were set at 22 rpm and 8°, respectively.

Purification of products was performed after 6 days of culture starting with a clarification step by depth filtration (Clarisolve 40MS, Merck) followed by bioburden reduction filter (Millipore Express SHF, Merck). Clarified broth was concentrated by tangential flow filtration (Hydrosart 30 KDa membrane, Sartorius) and then subjected to protein A affinity chromatography (MAb Select Sure, GE Healthcare) for complete mAb and HC purification or protein L for LC purification (Capto L, GE Healthcare), as described by the supplier. Purified fractions were buffer-exchanged through PD G-25 desalting columns, (GE). The final buffer was PBS for anti-HER2 and 50 mM citrate pH 6 for LC and HC.

### MAb denaturation and reduction

In order to obtain reduced and denatured mAbs chains, affinity purified trastuzumab at 5.75 mg/mL was buffer exchanged with a PD Desalting G-25 columns equilibrated with 0,584 M Tris-HCl (Roche, 10,708,976,001), and 5.37 mM EDTA (E6758, Sigma), pH 8.6 and then concentrated 5 fold using 30KDa Amicon tubes (Merck). Afterwards, 70 mM 2-mercaptoethanol (Bio-rad, 161–0710) and 8 M urea (Panreac, 141,754.1211) were added as described before [15]. The mixture was incubated for 1 h at 40 °C prior to the size-exclusion chromatography step.

### MAb chains physical separation

Trastuzumab chains were physically separated and purified by preparative SEC using an ÄKTA AVANT 150 system (GE) equipped with a 500 mL Superdex 200 XK 26/100 column (GE). The column was equilibrated with 0.584 M Tris-HCl, 5.37 mM EDTA, 7 mM 2-mercaptoethanol and 8 M urea at a flow rate of 3 mL/min. The column was kept at 4 °C in order to prevent cyanate formation due to the presence of urea. Peaks corresponding to trastuzumab HC or LC were separated and collected using the automated fraction collector. LC and HC structural integrity was assessed by performing a run with affinity chromatography, using HiScreen Capto L column (GE) for LC (binding buffer: sodium citrate 50 mM (Sigma, S1804), pH 6.5; elution buffer: sodium citrate 50 mM, pH = 2.3) and HiScreen Mab Select Sure (GE) for HC (binding buffer: 20 mM sodium phosphate, 150 mM NaCl, pH 7.4; elution buffer: sodium citrate 50 mM, pH 3.2).

### MAb or chains refolding by slow dialysis

Dialysis buffer: 3.6 mM 2-mercaptoethanol, 1.3 mM reduced glutathione (Sigma, G4251) and 1 mM EDTA in 0.1 M Tris-HCl, pH 8.

Feed initial composition: same as dialysis buffer adding 8 M urea.

**Fig. 8:**
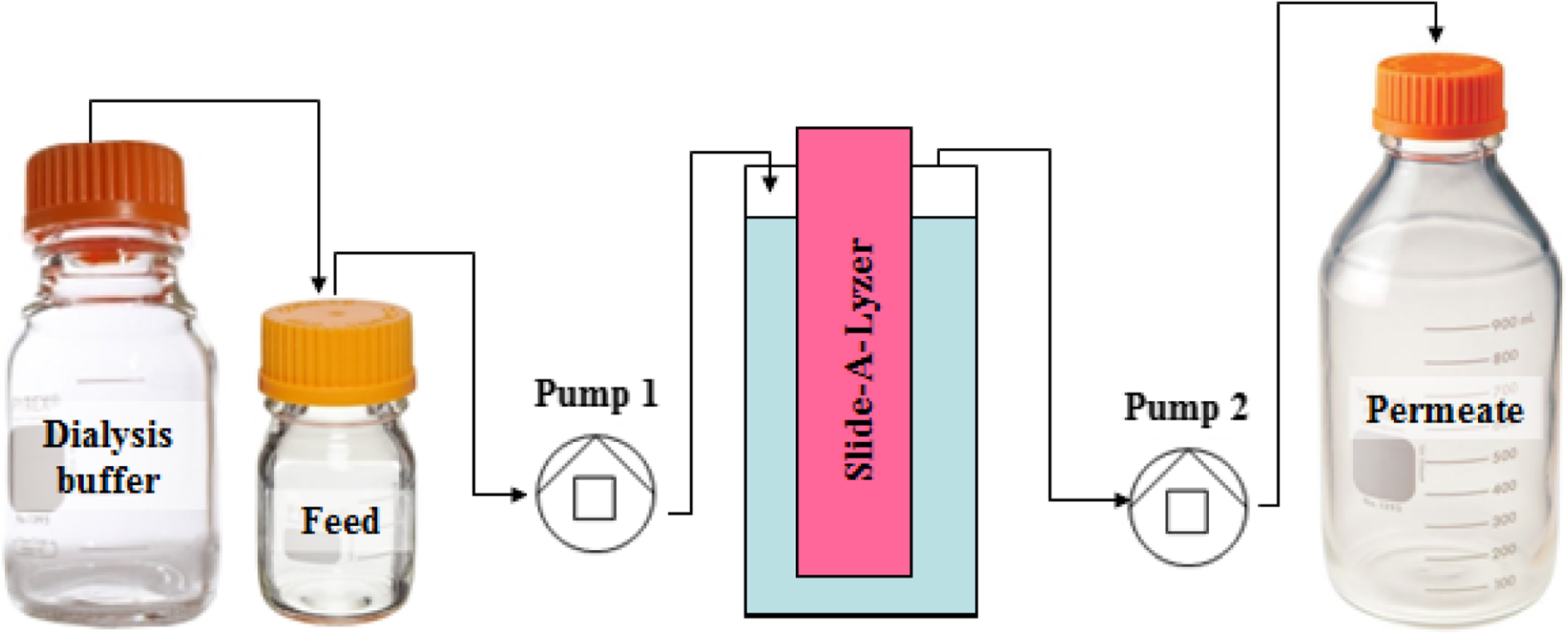
Slow dialysis scheme. The feed bottle was set in vacuum so the dialysis buffer feeds it at the same speed as the pump 1 feeds the reservoir. Pump 1 and pump 2 speed is 0,25 mL/min

Slow dialysis was performed by using Slide-A-Lyzer. Dialysis 3.5KDa (ThermoFisherScientific) device submerged in a 50 mL reservoir. The device contained the test sample plus 1 mL 8 M urea and 13.6 mg glutathione (Fig. 8), whereas the reservoir was filled with dialysis buffer and 8 M urea. Stirred feed bottle contained a constant volume of 50 mL of dialysis buffer, being constantly fed from dialysis bottle containing 400 mL of dialysis buffer.

A 0.25 mL/min constant flow rate was maintained during a 30 h process using a Schott Iberica peristaltic pump with 2 heads, during which the reservoir volume is maintained constant. Fig. 8 shows a scheme of the system.

All reagents used were acquired in Sigma-Aldrich unless otherwise noted.

### Analytical methods

#### SDS-PAGE gels

Protein mixture composition and relative amount of each species in the sample was analysed by denaturing, non-reducing SDS-PAGE (MiniProtean TGX Stain-free gels 4–20%, Biorad) following by band densitometry using GelDoc EZ software (Biorad).

#### SEC-HPLC

SEC analysis was performed in Waters Alliance 2695 (Waters) system using Zenix-C SEC-300 (4.6 × 300 mm, Sepax). The mobile phase was PBS (D1408, Sigma, diluted in ultrapure water up to 1x) at a flow rate of 0.35 ml/min. The UV absorbance was measured at a wavelength of 214 nm.

#### HIC-HPLC

HIC experiments were performed using a Waters Alliance 2695 system using Proteomix HIC Butyl-NP5 (4.6 × 50 mm, Sepax). The mobile phase was a gradient of 25 mM sodium phosphate pH 7 (S5136 and S5011, Sigma) with decreasing ammonium sulphate (2170, Merck) concentration (1.8 M to 0 M) and increasing concentration of isopropanol (AL03151000, Scharlab) to improve peaks resolution, as recommended by the column manufacturer, at a flow rate of 0.8 ml/min. The UV absorbance was measured at a wavelength of 214 nm.

#### ELISA assay

Antibody assembly was evaluated by comparing in vitro isolated anti-HER2 recognition with its in vivo assembled variant. Namely, 50 μL of 50 ng/μL HER2 in PBS solution (10004-HCCH-50, SinoBiological) was placed in the wells of 96-well Maxisorp plates (ThermoFisher Scientific) for its adsorption at 4 °C overnight. After removal of the supernatants, 100 μL of 2% skim milk (Oxoid, LP0031) in PBST (PBS plus 0.05% Tween 20 (Sigma, P2287)) were added and allowed to stand at room temperature for 1 h. The plates were washed three times with PBST and 50 μL of samples at different dilutions in PBS were placed in each well. After standing at room temperature for 1 h, the plates were washed three times with PBST. Then, 50 μL of a solution of 0.1 μg/mL of policlonal anti-human IgG1 conjugated to Horseradish peroxidase (Genscript, A10254) was added and the plates were allowed to stand at room temperature for 1 h. After three washings with PBST, 50 μL of TMB (Pierce, 1,854,050) were added. The staining reaction was carried out at room temperature for 10–15 min and stopped by adding 50 μL of 20% sulfuric acid (Panreac, 141,058.1611) addition. The absorbance at 450–630 nm was measured with a Labtech LT-4000 microplate reader (Labtech, MI, USA).

## Data Availability

The datasets used and/or analysed during the current study available from the corresponding author on reasonable request.

## Abbreviations

BiP: Immunoglobulin heavy chain binding protein
CH1: Constant Heavy chain subunit 1
CL: Constant Light chain
EDTA: Etilendiamintetraacetic acid
HC: Heavy Chain
HEK cells: Human Embryonic Kidney
Ig: Immunoglobulin
LC: Light Chain
mAb: Monoclonal antibody
ND: Not Detected
PDI: Protein Disulphide Isomerase
SDS-PAGE: Dodecyl sodium sulfate Polyacrilamide Electrophoresis Gel
